# Fatal childhood injuries in Finland between 1971 and 2017

**DOI:** 10.1186/s40621-020-00238-1

**Published:** 2020-04-06

**Authors:** Jari Parkkari, Ville M. Mattila, Seppo Niemi, Pekka Kannus

**Affiliations:** 1 Tampere Research Centre of Sports Medicine, UKK Institute for Health Promotion Research, P.O. Box 30, FIN-33501 Tampere, Finland; 2 Tampere University Hospital, Tampere, Finland; 3 University of Tampere, Tampere, Finland

**Keywords:** Fatal injury, Children, Epidemiology, Time trend

## Abstract

**Background:**

The injury mortality rates around the globe show considerable country-to-country differences and the rates have decreased at very different speeds. In Finland, the proportion of total mortality attributed to injuries has been one of the highest in the European Union. The purpose of the present study was to examine the 47-year nationwide trend and the male to female ratio in the number and incidence of fatal injuries among 0 to 14-year-old children in Finland.

**Methods:**

The data were obtained from the Official Cause-of-Death Statistics of Finland during 1971–2017. The main categories for unintentional injury deaths were road traffic injuries, water traffic injuries, falls, drownings, and poisonings. For intentional injury deaths, the main categories were suicides and homicides.

**Results:**

In 1971, there were 109 fatal injuries in girls and 207 in boys, while in 2017, these numbers had reduced to 9 and 12. The corresponding incidences (per 100,000 children per year) were 20.1 and 2.1 for girls, and 36.7 and 2.6 for boys, respectively. During the study period overall male to female injury incidence ratio decreased from 1.8 to 1.2. The greatest decline occurred in the number of fatal motor vehicle injuries. In 1971, the incidence of intentional deaths (suicides and homicides) was 2.6 in girls and 2.7 in boys, while in 2017 these numbers were 0.9 and 0.4, respectively.

**Conclusions:**

This nationwide study confirms that the number and incidence rates of childhood injury deaths have reduced till current days and are already below the average in Europe.

## Background

Childhood injuries are a major public health problem worldwide, injuries being by far the leading cause of death and disability for children from early childhood through adolescence (World Health Organization mortality database 2018; Corso et al. 2006; Xu et al. 2018; Kisser et al. 2017; Anderson et al. 2001). Child Environment Health Action Plan for Europe (CEHAPE) is an agreement where European Union member states in 2004 accepted to create and implement health and safety promotion programmes among children (http://www.childsafetyeurope.org/actionplans/index.html). In Finland, national CEHAP recommendations were published in 2007 (Erkkola et al. 2007). European countries have also been actively implementing recommendations of Child Safety Action Plan (CSAP) of European Child Safety Alliance (World Health Organization mortality database 2018; Anderson et al. 2001) (http://www.childsafetyeurope.org/actionplans/index.html). The Child Safety Action Plan initiative describes the process undertaken for the development of national child safety action plans in Europe. It highlights how the different phases of the project - assessment, strategic planning and action planning - fit together to support countries in their planning and cross-cutting approach to injury prevention (http://www.childsafetyeurope.org/actionplans/index.html).

The injury mortality rates in the Europe show considerable country-to-country differences and the rates have decreased at very different speeds (World Health Organization mortality database 2018; Kisser et al. 2017). In Finland, proportion of injury deaths of all deaths has been one of the highest in the European Union (Slätis and Ruusinen 1991; Ruusinen 1990). Maybe this is partly due to northern climate and lack of light during winter seasons. In 0 to 14-year-old Finnish children, the incidence of fatal injuries was 40 per 100,000 children in 1950, 30 in 1960, and 27 in 1970 (Slätis and Ruusinen 1991; Ruusinen 1990). and previously we reported the trend of childhood injury deaths in 1971–2010 (Parkkari et al. 2013). Thereafter, Finland has begun to address the areas of injury needing further attention in the 2009 Child Safety Report Card, the clearest development being creation of a national action plan (http://www.childsafetyeurope.org/actionplans/index.html). We have now monitored the trend of childhood injury deaths in Finland for 47 years (to the end of 2017) to assess most recent changes in the new millennium.

## Material and methods

We obtained from the Finnish Official Cause-of-Death Statistics (OCDS) the data for children aged 0–14 years who died because of an injury between 1971 and 2017. This statutory register has been computer-based since 1971, and from the beginning, it has been updated and quality-controlled by the Cause-of-Death Bureau at Statistics Finland (Official Statistics of Finland 2018a). The main categories for unintentional injury deaths are road traffic injuries, water traffic injuries, falls, drowning, and poisonings (Official Statistics of Finland 2018a). For intentional injury deaths, the main categories are suicides and homicides.

The coverage of the OCDS of Finland is in practice 100%, since each death, its certificate, and the corresponding person information in our computerized population register are cross-checked. The accuracy of the data is, in turn, maximized by a three-phase checking each code of the death certificate issued by the physician who certified the death (Official Statistics of Finland 2018a; Kannus et al. 1999). In injury-based deaths, the accuracy of the Finnish death certificates and their cause-of-death codes is verified further by autopsies performed in 94–97% of these deaths (Official Statistics of Finland 2018a; Kannus et al. 1999).

The mortality data were drawn from the entire 0 to 14-year-old children population of Finland, which was 1,107,280 in 1971 and 892,301 in 2017. Thus, the absolute numbers and incidences of deaths were not cohort-based estimates but true final results of the entire Finnish child population, and therefore, this study, similar to our previous epidemiologic investigations (Parkkari et al. 2013; Kannus et al. 1999), did not use statistical analyses with probability values and confidence intervals characteristically needed in cohort or sample-based estimations.

To establish age-specific incidences for the selected age-groups (under 1, 1 to 4, 5 to 9, 10 to 14), the annual numbers of fatal injuries were divided by the midyear population for each age- and sex-group. The annual midyear population figures for each age group between 1971 and 2017 were obtained from the Official Statistics of Finland, an official population register of the country (Official Statistics of Finland 2018b). In this statutory computer-based register, every Finn has been registered by her or his personal identification number and the register is continuously quality-controlled and updated by the Statistics Finland, the central statistical office of the country. The rates of fatal childhood injuries were expressed as the number of cases per 100,000 persons per year, by sex- and age-group.

Temporal trends in incidences of injury deaths were analysed by log-linear join point segmented regression models. Join point segmented regression analysis identified points where a statistically significant change over time in the linear slope of the trend occurred (Kim et al. 2000). The minimum / maximum number of join points was set to 0–3. The estimate annual percentage change (APC) for segmented analysis was reported. Join point analyses were performed using the Join Point Regression Program, (Version 4.2.0 - April 2015; Statistical Methodology and Applications Branch, Surveillance Research Program, National Cancer Institute).

## Results

**Fig. 1:**
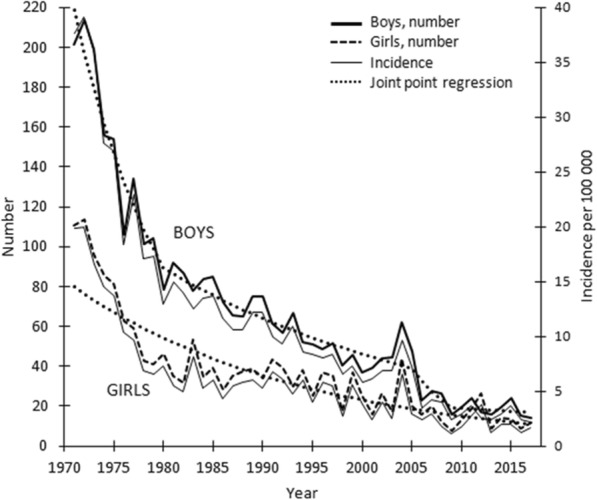
Annual number and incidence (per 100,000 persons) of fatal injuries among Finnish girls and boys aged 0 to 14 years between 1971 and 2017. Join point segmented regression analysis identified points where a statistically significant change over time in the linear slope of the incidence trend occurred: that is, years 1980, 2005 and 2009 in boys. In girls, no statistically significant trend change was observed

In 1971, 54% of all deaths of 1–14-year-olds were attributable to intentional or unintentional injuries; by 2017, this figure had decreased to 24%. Even more drastic decline was seen in the annual number and incidence of fatal injuries; they decreased continuously in both sexes (Fig. 1). In 1971, there were 109 fatal injuries in girls and 207 in boys, while in 2017, these numbers were 9 and 12. The corresponding incidences (per 100,000 children per year) were 20.1 and 2.1 for girls and 36.7 and 2.6 for boys. The relative decreases were 90% for girls and 93% for boys. Over the study period, the overall male female ratio decreased from 1.83 to 1.24. Between 1971 and 1980, the deceasing trend in the rate of boys’ fatal injuries was clear with an annual percentage change (APC) of − 9.5% (Fig. 1). Between the years 1980 and 2005, this decline was slower with APC of − 3.2%. Between 2005 and 2009 APC was − 16.2% and between 2009 and 2017–1.7%, respectively. In girls, there was no statistically significant change in the slope of the incidence trend. Annual percentage change was − 4.2% over the whole 47-year-period (Fig. 1).

Age-specifically, the incidence (per 100,000 persons per year) of fatal injuries decreased considerably in all age groups (under 1, 1 to 4, 5 to 9, 10 to 14) during the 47-year follow-up period (Table 1). 10–14-year-old children had the highest number of fatal injuries. In 2017, the incidence (per 100,000 persons per year) among 10–14-year-old girls was 3.4 and 3.9 in boys (Table 1). Male female ratio in this age group was 1.15.

**Table 1:**
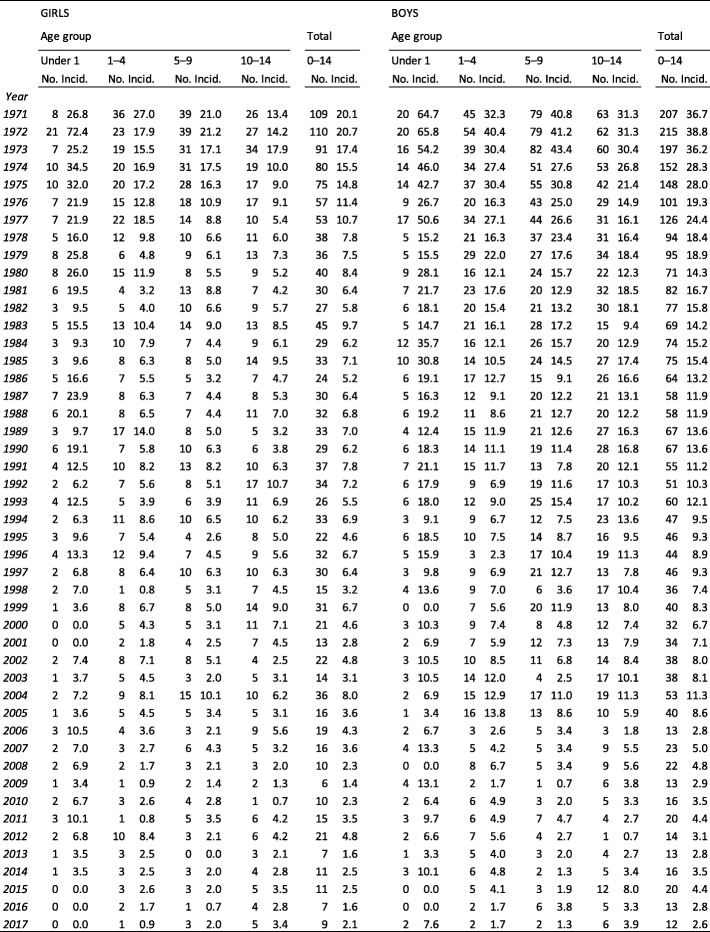
Numbers and age-specific incidences (per 100,000 persons) of injury-induced deaths in Finnish girls and boys between 1971 and 2017

The observed general reduction in fatal injuries was mostly due to decreasing number of unintentional injuries. The greatest decline occurred in the number of fatal motor vehicle injuries: from 57 (girls) and 92 (boys) in 1971 to 2 (girls) and 5 (boys) in 2017. However, the trend was decreasing in violence-related deaths, too. In 1971, there were 14 intentional deaths in girls and 15 in boys, while in 2017, these numbers were 4 and 2, respectively. The corresponding incidences (per 100,000 children per year) were 2.6 and 0.9 for girls, and 2.7 and 0.4 for boys.

In 1971, 48% of all the injury deaths among 0–14-year-old Finnish children were motor vehicle injuries, 17% were drownings, and 9% intentional injuries. In 2017, the corresponding percentages were 38, 10 and 29%, respectively.

## Discussion

We studied all Finnish children 14 years of age or younger to describe the trends of fatal injuries between 1971 and 2017. The findings confirm that there has been a decline in pediatric injury deaths over the years studied and this development has continued till current days (less than 3 fatal injuries per 100,000 children per year). The number and incidence of fatal injuries in boys have decreased almost to the level of girls.

A World Health Organization (WHO) mortality database shows that the incidence of injury-related deaths in children under 14 has been declining in the OECD (Organisation for Economic Co-operation and Development) nations in 1971–2017 (World Health Organization mortality database 2018). The decline in many European Union countries gives good evidence that most of the fatal injuries can be prevented (World Health Organization mortality database 2018; Kisser et al. 2017). Our results are in accordance with those reported from other western countries. Road and other unintentional injuries show a general decrease, whereas the pattern for violence-related deaths varies between countries. Moreover, the gap between girls and boys has reduced in many countries (World Health Organization mortality database 2018; Kisser et al. 2017; Pressley et al. 2007; Ekman et al. 2005; Pearson et al. 2009) In the present study suicides and homicides were already more common in girls than boys in 2017.

In Finland, injuries in children has been a well-recognized public health problem over a period of six decades (Slätis and Ruusinen 1991; Ruusinen 1990; Parkkari et al. 2013). In 0 to 14-year-old Finnish children, the incidence of fatal injuries was 40 per 100,000 children in 1950, 30 in 1960, and 27 in 1970 (Slätis and Ruusinen 1991; Ruusinen 1990). The present study showed that the number and incidence rates of childhood injury deaths have reduced till current days and are already below the average in Europe (less than 3 fatal injuries per 100,000 children per year) (Kisser et al. 2017). The most recent changes in the new millennium are important, because not only speculations about the reasons behind the changes but also facts about the fresh annual numbers of adolescent injury deaths are of interest. The reasons for the positive development are multifactorial, but the most obvious explanation is the improved traffic safety, perhaps as the result of the multifaceted traffic safety program conducted in Finland since late 1960s. The program has included serious efforts for road and traffic planning and legislation, more comprehensive traffic supervision and control (particularly controlling speeds and driving under the influence of alcohol or illicit drugs), improved vehicle safety (car body, seats, seat belts, and child safety seat restraints) and intense promotion of bicycle helmets. Environmental measures such as improving highway networks have probably conferred a positive impact. Also, education towards increased general awareness of adolescents’ high-risk situations (for example, increased education on safety routes when commuting to school and hobbies) are probably among the most important single factors.

An additional factor that may relate to the above noted declining time trend in fatal injuries is more easily accessible emergency services and improved trauma care. The national registry of road traffic accidents shows that total number of traffic crashes in Finland has been declining in 1971–2016, although the number of vehicles and driven kilometres have increased considerably during that time. In 1971 there were 30,005 traffic accidents in Finland, in 2016 this figure was 20,343 (Traffic accidents in Finland in 1931-2016 n.d.). At the same time points the number of injured persons was 10,424 and 4513, respectively. In 1971 10.0% of these injured persons died while in 2016 this figure fell to 5.3%. This indicates that an additional factor that may relate to the above noted declining secular trend in fatal injuries is the improved accessibility to emergency services and improved trauma care. EU funded SafetyNet project has developed road safety performance indicators which characterize the level of trauma management systems performance in European countries. These indicators enable country comparisons and facilitate national development of trauma care services and facilities (Gitelman et al. 2013).

Concerning prevention of fatal home and leisure time injuries education towards increased general awareness of children’s high-risk situations, safer play grounds, child-resistant packaging and keeping poisonous substances out of reach of children are probably among the most important single factors. Drowning is a leading cause of injury-death in children in many areas (Gardner et al. 2010). Whereas Finland has a long sea coastline and more than 200.000 lakes the number of drownings reached the ultimate goal of injury prevention in 2017, i.e. there were zero drownings in Finnish 0 to 14-year-old girls. The reasons for the positive development are multifactorial, but the most obvious explanation is the education and advocates towards increased swimming skills and general awareness of children’s high-risk situations near water (Lunetta et al. 2004; Franklin et al. 2017).

Intentional injury (violence) as a cause of injury has received much attention in Finland during recent years especially because of increasing number of hospitalizations due to alcohol poisonings among adolescent population (Kivistö 2009). In the present study, intentional injuries accounted for 29% of the total child injury-mortality in 2017. Although the total number of suicides and homicides in children is not high, there is a continuous need for preventive measures to avoid such deaths among adolescent population. Particular attention is needed to address injury prevention related to the use of alcohol, medications and other drugs. Further work is required to prevent injuries occurring in and around the home and during leisure time. The majority of injuries in children actually come from sports but they are seldom fatal (Kisser et al. 2017; Räisänen et al. 2018). It is clear from the Finnish child safety scores that although progress has been made more can be done in policy introduction and implementation, and in enforcement of evidence-based intervention strategies to prevent sports injuries (Kisser et al. 2017; Räisänen et al. 2018). There is also a need to support and fund sports injury prevention measures using a combined approach of education, engineering and enforcement of standards and regulations.

A major strength of this study is that the data of deaths was drawn from the nationwide Official Cause-of-Death Statistics of Finland, a database with a high accuracy and excellent coverage. Finnish law dictates that all victims of a sudden, unexpected death have to be autopsied to confirm to cause of death, and, in practice autopsy is performed in 94–97% of these cases (Official Statistics of Finland 2018a; Kannus et al. 1999). Strength was also that the annual midyear population figures in 1971–2017 were obtained from the Official Statistics of Finland, a statutory computer-based population register of the country (Official Statistics of Finland 2018b). The described trends in the absolute numbers and incidences of injuries were thus not cohort-based estimates but true nationwide results.

The limitation of the study is that it provides information only from one European Union country and due to wide diversity of preventive measures and programmes in different sectors it is difficult to evaluate which actions have been the most effective. Randomised clinical trials are not feasible in most categories of fatal injuries because these incidents are so rare in nature. Limitations is also the fact that we do not exactly know in what extent the trends that are evident in this paper are indicative of improved safety of environment e.g. cars or whether trauma care has improved. However, as discussed above, the national traffic accident registry indicates that both these factors are important behind the development (Traffic accidents in Finland in 1931-2016 n.d.). It is also clear from the Finnish child safety scores that progress has been made by following the action plan (http://www.childsafetyeurope.org/actionplans/index.html). Moreover, firearm-related deaths can cause significant differences between countries (Fowler et al. 2017). The same concerns countries and areas where downhill skiing is a popular hobby, since skiing-related collisions and severe head injuries are known to cause fatalities among children (Kisser et al. 2017; Xiang et al. 2004). The wide variation of unintentional injury mortality rates in the European Union member states suggests that there is still high potential for injury prevention. Thousands of lives could be saved annually if all countries achieve the level of the lowest national injury mortality rates in the EU (World Health Organization mortality database 2018; Kisser et al. 2017).

## Conclusions

The decreasing trend of fatal childhood injuries has continued in Finland till 2017. The number and incidence of fatal injuries in boys have decreased almost to the level of girls. In intentional deaths boys’ numbers are now even lower than that of girls. To keep the numbers of these children’s unintentional and intentional injuries low, continuous nationwide regulatory actions, environmental modifications, and educational measures are needed.

## What is already known on the subject

- Childhood injuries are a major public health problem worldwide, injuries being by far the leading cause of death and disability from early childhood through adolescence.
- In Finland, the frequency of fatal childhood injuries has been one of the highest in the western Europe. In 0 to 14-year-old Finnish children, the incidence of fatal injuries was 40 per 100,000 children in 1950, 30 in 1960, 27 in 1970 and 3 in 2010.

## What this study adds

- The present study showed that positive development of childhood injury deaths has continued till current days (less than 3 fatal injuries per 100,000 children per year), the greatest decline occurring in the number of fatal motor vehicle and intentional injuries.
- The gap in fatal injuries between girls and boys has continuously reduced.
- In intentional deaths (suicides and homicides) boys’ numbers are now lower than that of girls.
- The wide variation of unintentional injury mortality rates in the European Union member states suggests that there is still high potential for injury prevention. Thousands of lives could be saved annually if all countries achieve the level of the lowest national injury mortality rates.

## Data Availability

All data generated or analysed during this study are included in this published article [and its supplementary information files].

## References

[CR1] Anderson M, Kaufman J, Simon TR, Barrios L, Paulozzi L, Ryan G, Hammond R, Modzeleski W, Feucht T, Potter L (2001). School-associated violent deaths study group. School-associated violent deaths in the United States, 1994-1999. JAMA.

[CR2] Corso P, Finkelstein E, Miller T, Fiebelkorn I, Zaloshnja E (2006). Incidence and lifetime costs of injuries in the United States. Inj Prev.

[CR3] Ekman R, Svanström L, Långberg B (2005). Temporal trends, gender, and geographic distributions in child and youth injury rates in Sweden. Inj Prev.

[CR4] Erkkola M, Fogelholm M, Huuskonen MS (2007). Lasten ympäristö ja terveys. National CEHAP-plan (in Finnish).

[CR5] Fowler KA, Dahlberg LL, Haileyesus T, Gutierrez C, Bacon S. Childhood Firearm Injuries in the United States. Pediatrics. 2017;140(1). 10.1542/peds.2016-3486. 10.1542/peds.2016-3486 PMC6488039 28630118

[CR6] Franklin RC, Pearn JH, Peden AE (2017). Drowning fatalities in childhood: the role of pre-existing medical conditions. Arch Dis Child.

[CR7] Gardner HG, Baum CR, Dowd MD, Durbin DR, Ebel BE, Lichenstein R, Limbos MA, O'Neil J, Quinlan KP, Scholer SJ, Sege RD, Turner MS, Weiss J (2010). Prevention of drowning. American Academy of Pediatrics Committee on Injury, Violence, and Poison Prevention. Pediatrics.

[CR8] Gitelman V, Auerbach K, Doveh E (2013). Development of road safety performance indicators for trauma management in Europe. Accid Anal Prev.

[CR9] Kannus P, Parkkari J, Koskinen S, Niemi S, Palvanen M, Järvinen M, Vuori I (1999). Fall-induced injuries and deaths among older adults. JAMA.

[CR10] Kim HJ, Fay MP, Feuer EJ, Midthune DN (2000). Permutation tests for joinpoint regression with applications to cancer rates. Stat Med.

[CR11] Kisser R, Walters A, Rogmans W, Turner S, Lyons RA (2017). Injuries in the European Union 2013–2015.

[CR12] Kivistö J (2009). Poisonings in Finnish Children. Doctoral dissertation. Acta Universitatis Tamperensis 1378.

[CR13] Lunetta P, Smith GS, Penttilä A, Sajantila A (2004). Unintentional drowning in Finland 1970–2000: a population-based study. Int J Epidemiol.

[CR14] Official Statistics of Finland (2018). Official Cause-of Death Statistics 2017.

[CR15] Official Statistics of Finland (2018). Population Structure 1970-2017.

[CR16] Parkkari J, Mattila V, Kivistö J, Niemi S, Palvanen M, Kannus P (2013). Fatal childhood injuries in Finland, 1971–2010. Inj Prev.

[CR17] Pearson J, Jeffrey S, Stone DH (2009). Varying gender pattern of childhood injury mortality over time in Scotland. Arch Dis Child.

[CR18] Pressley JC, Barlow B, Kendig T, Paneth-Pollak R (2007). Twenty-year trends in fatal injuries to very young children: the persistence of racial disparities. Pediatrics.

[CR19] Räisänen AM, Kokko S, Pasanen K, Leppänen M, Rimpelä A, Villberg J, Parkkari J (2018). Prevalence of adolescent physical activity-related injuries in sports, leisure time, and school: the National Physical Activity Behaviour Study for children and Adolescents. BMC Musculoskelet Disord.

[CR20] Ruusinen A (1990). Lasten tapaturmatilanne (in Finnish). Suomen Lääkärilehti (Finnish Medical Journal).

[CR21] Slätis P, Ruusinen A (1991). Orthopedic diseases and trauma in Finland. Trends in consumption of health services 1970-1985. Acta Orthop Scand Suppl.

[CR22] Traffic accidents in Finland in 1931-2016 (n.d.). https://www.stat.fi.

[CR23] World Health Organization mortality database 2018. https://www.who.int/healthinfo/mortality_data/en/.

[CR24] Xiang H, Stallones L, Smith GA (2004). Downhill skiing injury fatalities among children. Inj Prev.

[CR25] Xu J, Murphy SL, Kochanek KD, Bastian B, Arias E (2018). Fatal injuries among children. Centers for Disease Control and Prevention (CDC). Division of vital statistics. Natl Vital Stat Rep.

